# A policy agenda to support care partners of people living with ADRD from historically underserved communities

**DOI:** 10.1002/alz.086494

**Published:** 2025-01-09

**Authors:** Sherril B Gelmon, Walter D Dawson, Jenn Hollandsworth Reed

**Affiliations:** ^1^ OHSU‐PSU School of Public Health, Portland, OR USA; ^2^ GBHI Lived Experience Panel, Portland, OR USA; ^3^ Global Brain Health Institute, San Francisco, CA USA; ^4^ Oregon Health & Science University, Portland, OR USA; ^5^ Portland State University, Portland, OR USA

## Abstract

**Background:**

Alzheimer’s disease and related dementias (ADRD) place enormous burdens on families, care partners, and the public programs that finance ADRD services. Caregiving is disproportionately provided by women, individuals of low socioeconomic status, and underserved minority populations, who also rely upon informal care and support. National dementia strategies rarely address the needs of care partners. This study was designed to identify policy opportunities that would emphasize and address care partner needs, with an emphasis on historically and currently underserved communities.

**Method:**

Twenty‐four (n = 24) systematic interviews and five focus groups (n = 5) were conducted with care partners and organizations offering services that support people with ADRD. Data collection focused on historically underserved communities in the United States. Organizational leaders and care partners also participated in a modified Delphi process to identify priorities for supporting care partners. A comprehensive literature review provided additional information.

**Results:**

Care partners from all populations reported lacking supports to help manage caregiving, despite being generally satisfied with clinical care and research involvement. Specific needs and types of supports varied across populations, due to cultural traditions, multiple systemic inequities, and legacies of structural racism. The findings suggest multiple unmet care partner needs across all populations, including ADRD‐specific information; navigation and coordination within and across systems; access to clinicians, care teams, and health/personal records; legal and financial resources; psychological and physical health supports; and in‐home and respite care.

These findings suggest policy opportunities to address specific needs of ADRD care partners. While the findings are derived from the US context, they have relevance for other national dementia strategies. Policy recommendations were identified related to equity of access, expansion of public insurance, workforce development, creation of information hubs, and adoption of technologies. Policies should support care partners by incorporating incentives for care coordination and navigation.

**Conclusion:**

Changes in health and social policies are needed to better support care partners of people living with ADRD. Policymakers must collaborate with care partners to develop and implement new national dementia strategies to improve care partner experiences. To ensure equitable service provision, culturally appropriate policies and supports for care partners from underserved communities require prioritization.

